# Mitochondria as Nutritional Targets to Maintain Muscle Health and Physical Function During Ageing

**DOI:** 10.1007/s40279-024-02072-7

**Published:** 2024-07-26

**Authors:** Sophie C. Broome, Jamie Whitfield, Leonidas G. Karagounis, John A. Hawley

**Affiliations:** 1 Exercise and Nutrition Research Program, Mary MacKillop Institute for Health Research, Australian Catholic University, Melbourne, VIC 3000 Australia; 2 Institute of Social and Preventive Medicine (ISPM), University of Bern, Bern, Switzerland

## Abstract

The age-related loss of skeletal muscle mass and physical function leads to a loss of independence and an increased reliance on health-care. Mitochondria are crucial in the aetiology of sarcopenia and have been identified as key targets for interventions that can attenuate declines in physical capacity. Exercise training is a primary intervention that reduces many of the deleterious effects of ageing in skeletal muscle quality and function. However, habitual levels of physical activity decline with age, making it necessary to implement adjunct treatments to maintain skeletal muscle mitochondrial health and physical function. This review provides an overview of the effects of ageing and exercise training on human skeletal muscle mitochondria and considers several supplements that have plausible mechanistic underpinning to improve physical function in ageing through their interactions with mitochondria. Several supplements, including MitoQ, urolithin A, omega-3 polyunsaturated fatty acids (n3-PUFAs), and a combination of glycine and N-acetylcysteine (GlyNAC) can improve physical function in older individuals through a variety of inter-dependent mechanisms including increases in mitochondrial biogenesis and energetics, decreases in mitochondrial reactive oxygen species emission and oxidative damage, and improvements in mitochondrial quality control. While there is evidence that some nicotinamide adenine dinucleotide precursors can improve physical function in older individuals, such an outcome seems unrelated to and independent of changes in skeletal muscle mitochondrial function. Future research should investigate the safety and efficacy of compounds that can improve skeletal muscle health in preclinical models through mechanisms involving mitochondria, such as mitochondrial-derived peptides and mitochondrial uncouplers, with a view to extending the human health-span.

## Key Points

**Table:** 

Skeletal muscle mitochondria are key targets for interventions that can attenuate age-related declines in muscle mass and physical function.
Exercise training counteracts many of the deleterious effects of ageing on skeletal muscle mitochondria. However, complementary interventions are needed as many older adults fail to meet physical activity guidelines.
Several supplements, including MitoQ, urolithin A, n-3 PUFAs and GlyNAC improve physical function in older individuals through mechanisms involving mitochondria, including increases in mitochondrial biogenesis and bioenergetics, decreases in mitochondrial ROS emission and oxidative damage, and improvements in mitochondrial quality control.

## Introduction

The number of adults aged 65 years or older globally is predicted to increase more than twofold by 2050, reaching an estimated 1.6 billion, or one in every six persons alive [1]. While global life expectancy has increased during the past three decades, health-span (the length of time a person remains healthy) has lagged, with older adults (greater than 65 years) now likely to live their last decade in poor health [2]. At the population level, ageing will have a significant economic impact as a reduced labour force and associated reduction in tax revenue, coupled with a greater demand for government programs that support older individuals, will place unsustainable fiscal pressure on government budgets. In Australia (population 25 million), this is projected to equate to an annual cost of $36 billion by 2028–29 [3]. Consequently, there is an urgent need to focus research resources on cost-effective strategies that contribute to the maintenance of functional longevity.

One of the most notable morphological changes observed with ageing is a progressive loss of skeletal muscle mass and quality, which is accompanied by a deterioration in physical function [4]. Age-related declines in muscle mass and function (i.e., sarcopenia) are related to reductions in the ability to undertake tasks of daily living [5], an increased risk of falls and fractures [6], and numerous metabolic impairments [7], ultimately leading to a cascade of events that precipitate a loss of physical independence and an increased reliance on care. Age-associated changes in skeletal muscle mitochondrial content, bioenergetics, reactive oxygen species (ROS) emission, and quality control have been identified and studied as putative candidates underpinning the loss of muscle mass and physical function that occurs with ageing [8, 9]. As such, several of these factors may be key targets for interventions that can attenuate declines in muscle structure and function.

To date, pharmacological interventions to combat sarcopenia have failed to produce clinically relevant outcomes regarding improvements in strength and physical performance. As such, exercise training is still arguably the most potent, cost-effective, and practical intervention for the preservation of muscle mass and function for most of the population throughout adult life. However, levels of habitual physical activity progressively decline with increasing age, and the majority of older adults fail to meet the physical activity guidelines outlined by the World Health Organisation [10]. As a result, alternative interventions that improve mitochondrial function and promote functional longevity independently or in combination with exercise are required. Other lifestyle interventions that have been shown to impact mitochondria include sleep [11], periods of energy restriction [12], and nutritional interventions such as supplements. This review summarises the effects of ageing and exercise training on human skeletal muscle mitochondria and discusses novel complementary supplement interventions that have plausible mechanistic underpinning to improve physical function in ageing through their interactions with mitochondria.

## Mitochondria and Ageing Skeletal Muscle: Use It or Lose It

Ageing results in a decline in cardiovascular fitness (measured as an individual’s maximal aerobic capacity (V̇O_2_ max) beginning in the third decade of life [13]. While age-related declines in maximal aerobic capacity are largely related to reductions in maximal cardiac output [14], they may also be linked to age-related loss of muscle mass and declines in mitochondrial content and/or function. Beyond adenosine triphosphate (ATP) production, mitochondria play important roles in multiple cellular processes such as calcium uptake and extrusion, iron-sulphur cluster synthesis, and mitochondrial DNA (mtDNA) maintenance and expression [15]. Mitochondria also act as systemic signalling hubs that transduce information within and between cells [17]. For a comprehensive summary of known mitochondrial functions, readers are referred to several recent reviews [15–17].

Experimental evidence from mice and human studies suggest that mitochondria are crucial in the aetiology of sarcopenia [18]. Specifically, dysregulation in mitochondrial turnover via the mitochondrial-specific form of autophagy, mitophagy, is linked to elevated mitochondrial ROS emission and the activation of proteolytic pathways responsible for muscle protein breakdown and ‘anabolic resistance’ [19]. Muscle of older individuals also exhibits substantial loss of neuromuscular junction integrity and loss of innervation resulting in increased mitochondrial ROS emission in the denervated fibres and neighbouring innervated fibers in the same muscle [20, 21]. Age-related declines in mitochondrial bioenergetics (e.g., mitochondrial capacity and efficiency) have been proposed as a factor underpinning low physical function [22, 23], fatiguability [24], and loss of exercise efficiency (defined as the ratio of mechanical work rate over energy expenditure) with ageing [25]. Furthermore, there is some evidence that age-related changes in mitochondrial positioning within the muscle away from the calcium release units on the sarcoplasmic reticulum may underlie fatigue due to disruptions in calcium handling [26]. Despite many years of research investigating potential associations between mitochondria and ageing, there exists considerable debate as to the direct role of mitochondrial ‘dysfunction’ in age-related declines in muscle mass and function. This, in part, is because it is difficult to isolate the direct effects of ageing per se on mitochondria from the effects of low levels of physical activity, since these events are inter-related (i.e., age-related changes in mitochondrial content can contribute to a decline in physical activity and vice versa). Indeed, the age and level of habitual physical activity of a cohort under investigation can be a major confounder when interpreting the impact of a given intervention on mitochondrial content and/or function. Deeper investigation of the mitochondrial changes that accompany ageing is therefore crucial to understand how mitochondria might be targeted to promote the maintenance of physical function. Given that skeletal muscle contains highly specialised and metabolically distinct mitochondrial subpopulations [27, 28], future research investigating the effects of ageing and exercise on mitochondria should also take into account the independent pathways of these distinctly different populations.

### Mitochondrial Content and Plasticity

One of the most inconsistent findings in the literature concerns the impact of ageing on skeletal muscle mitochondrial content, with some studies reporting no change in mitochondrial content in individuals aged over 65 years compared to their young counterparts [29–31] and others observing a decrease [32–34]. Such differences may, in part, be related to the various measures used to determine mitochondrial content, including maximal enzyme activities, mitochondrial protein levels, and mitochondrial density/volume. Mitochondrial content is typically up- or down-regulated based on the metabolic activity of the tissue/organ under investigation. In the context of exercise training, mitochondrial adaptations are largely dependent on the frequency, intensity and duration of an individual’s habitual activity levels [35, 36]. As such, the divergent levels of physical activity and exercise patterns that often accompany ageing are likely to contribute to the inconsistent results between investigations. Studies involving activity-matched old and young participants show little or no decline in several indices of mitochondrial content in ageing muscle [29]. Furthermore, comparisons between elderly subjects differing in training status demonstrate that lifelong training maintains the expression and activity of proteins associated with oxidative metabolism like those observed in muscle of young individuals [37, 38]. Whether differences in physical activity status alone can explain the discordant results remains to be determined.

Irrespective of age, mitochondria have been shown to respond similarly to an acute exercise challenge and exercise training in both young and old individuals, with similar exercise-induced mitochondrial signalling responses observed in skeletal muscle of older compared with younger males, independent of prior training status [39]. This is consistent with data indicating normal mitochondrial remodelling responses to long-term exercise training in the elderly [40, 41]. Therefore, it appears that ageing is not accompanied by diminished mitochondrial plasticity in response to both acute and chronic exercise stimuli [42].

### Mitochondrial Bioenergetics

Considerable discussion surrounds the concept of decreased mitochondrial capacity with ageing. Several cross-sectional studies have demonstrated decreased mitochondrial capacity with advancing age [34, 40, 41, 43–45], although this finding is not universal [29, 46–49]. Such variability may be observed because many studies fail to consider important covariates, such as physical activity levels, cardiorespiratory fitness [43], and adiposity [44], which are likely to confound the relationship between mitochondrial capacity and age. Several studies have also used isolated mitochondria preparations [34, 45, 50], which tend to amplify age-related deficits in mitochondrial function compared to measurements conducted on permeabilized myofibers where the mitochondrial reticulum is maintained [51]. Contemporary research has shown that maximal mitochondrial respiration is not influenced by chronological age per se but is closely related to cardiorespiratory fitness and body composition [52], suggesting the aetiology of lower mitochondrial bioenergetics in ageing muscle may be primarily due to low levels of physical activity. Indeed, exercise training preserves mitochondrial capacity in older individuals when measured in both isolated mitochondria and permeabilized myofibers [40, 41], with several studies reporting no differences in mitochondrial capacity when young and old participants are matched for physical activity levels, V̇O_2_ max and body mass index (BMI) [29, 48, 53].

While maximal mitochondrial respiration does not appear to be largely influenced by ageing, a recent study reported that ageing is associated with a decline in skeletal muscle mitochondrial respiration in the presence of non-saturating adenosine diphosphate (ADP) concentrations that more closely reflect the in vivo metabolic milieu [46]. Furthermore, the ability of ADP to stimulate respiration (i.e., apparent sensitivity or K_m_) was also decreased in skeletal muscle of older individuals [46]. This is consistent with previous work in rodents, and suggests the underlying cause of mitochondrial defects in ageing muscle may be due to dysregulation of the adenosine nucleotide translocator (ANT), which transports ADP into the mitochondrial matrix [54]. Crucially, in that study, resistance exercise training reversed the age-related derangements in mitochondrial ADP sensitivity, further highlighting the capacity for exercise training to improve or potentially preserve some aspects of mitochondrial bioenergetics that occur with ageing [46].

### Mitochondrial Reactive Oxygen Species Emission

Mitochondrial ROS play important roles in cellular signalling and in the promotion of several beneficial cellular adaptations to stress [55]. However, oxidative stress has been implicated in age-related muscle atrophy [19], suggesting there is a ‘threshold’ beyond which further elevations in ROS levels become deleterious. The notion that increased mitochondrial ROS production contributes to ageing in humans is a current topic of lively scientific debate [56–58]. Studies using isolated mitochondria report increased maximal mitochondrial H_2_O_2_ release with ageing [59]. However, isolating mitochondria potentiates ROS emission [60] and exaggerates the impact of ageing [51]. In humans, the maximal capacity for mitochondria to produce ROS is not increased with ageing when assessed using methods that preserve mitochondrial structure [29, 46]. In contrast, muscle immobilisation induces an increase in mitochondrial ROS in otherwise healthy individuals [61, 62], suggesting regular physical activity may be an effective way of attenuating increases in mitochondrial ROS in ageing skeletal muscle.

The transport of ADP into mitochondria and the subsequent binding of ADP to ATP synthase decreases membrane potential and the overall rate of mitochondrial superoxide production while simultaneously stimulating aerobic metabolism. While maximal rates of mitochondrial H_2_O_2_ emission are unaltered with ageing, the ability of ADP to reduce mitochondrial ROS emission is impaired in older individuals, leading to elevated mitochondrial ROS emission at submaximal ATP concentrations [46]. Resistance-based exercise training reverses age-related declines in mitochondrial ADP sensitivity while concurrently increasing the capacity for mitochondrial H_2_O_2_ emission, possibly through the induction of mitochondrial biogenesis [46]. Consequently, age-related increases in mitochondrial H_2_O_2_ emission in the presence of non-saturating ADP concentrations are not impacted by resistance exercise training [46]. Short-term resistance exercise training has also been shown to increase mitochondrial ROS emission during recovery from muscle immobilisation [62], suggesting that resistance training does not restore perturbations of mitochondrial redox homeostasis. Future work should investigate the ability of aerobic-based exercise training to improve ADP sensitivity and restore redox balance in aged muscle as this type of training may reverse immobilisation-induced increases in mitochondrial ROS emission [61].

### Mitochondrial Quality Control

The mitochondrial reticulum is a dynamic structure undergoing constant remodelling. Mitochondrial fusion allows mitochondrial components to be exchanged and diluted while mitochondrial fission segregates damaged mitochondria for removal by mitophagy. These processes are essential for the maintenance of a healthy, functioning mitochondrial network. Several studies have investigated whether mitochondrial quality control is influenced by ageing, with inconclusive results. While some investigations report no age-related differences in the skeletal muscle expression of the major regulators of mitochondrial fusion (optic atrophy-1 (OPA1), mitofusin 1 (MFN1) and mitofusin 2 (MFN2)) and fission (mitochondrial fission 1 protein (FIS1), dynamin-related protein 1 (DRP1)) [22, 52, 63, 64], others find upregulation of mitochondrial dynamics proteins MFN2 and mitochondrial dynamics protein 49 (MiD49) in aged skeletal muscle, with the increased abundance of MFN2 exclusive to type II (‘fast twitch’, glycolytic) muscle fibres [30]. In that study [30], high-intensity interval training reversed the increase in MFN2 expression in type II fibres. Upregulation of MFN2 and MiD49 with ageing may be a protective mechanism against mitochondrial dysfunction, particularly in type II muscle fibre. In this context, exercise, particularly resistance-based training, may have a unique protective effect in negating the need for increased turnover of mitochondria. Therefore, age-related changes in mitochondrial quality control are likely related to low levels of habitual physical activity.

The effect of ageing on mitophagy is poorly understood. Decreased expression of mitophagy genes BNIP, DRP1 and Parkin has been observed in skeletal muscle of physically inactive elderly women [65]. In contrast, no differences in skeletal muscle Parkin protein expression between young and old physically active individuals have been reported [29]. To date, studies investigating the effect of ageing on mitophagy have focused on static measures of mRNA and protein in whole-cell lysates, which may be inadequate to capture and accurately quantify this dynamic process or elucidate what is happening at the mitochondrial level [29]. As a result, the impact of ageing on mitophagy is unclear.

**Fig. 1:**
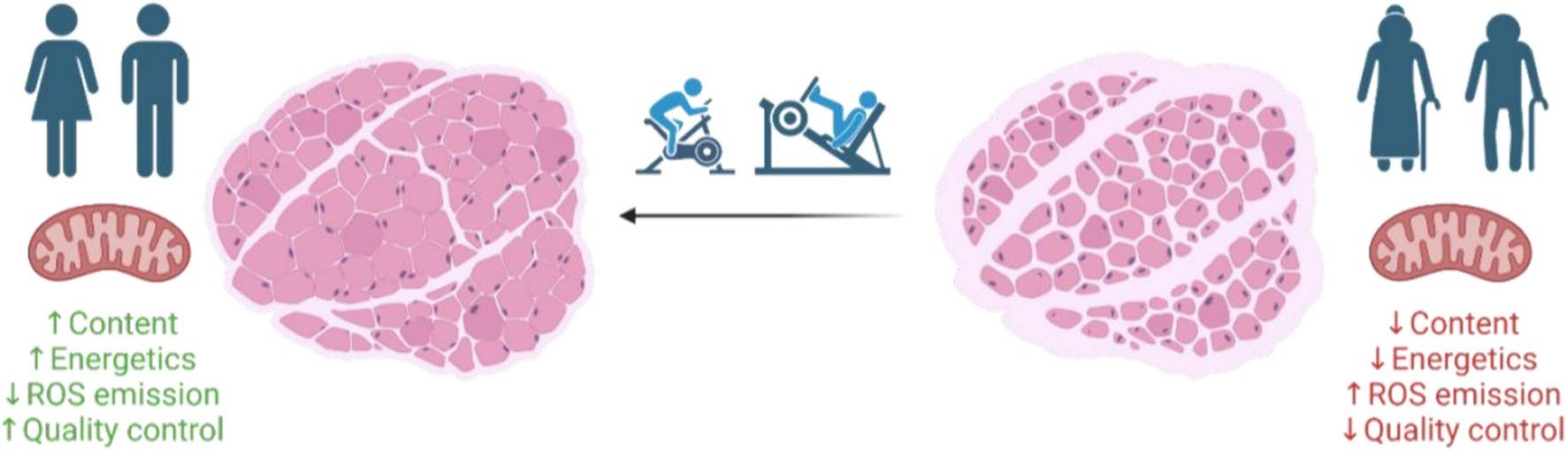
Exercise training attenuates many of the deleterious effects of ageing on mitochondria. Exercise training restores mitochondrial content, energetics, ROS emission, and quality control in aged skeletal muscle leading to improved muscle mass and physical function. *ROS* reactive oxygen species

In summary, exercise training can attenuate, and in some cases reverse, many of the deleterious effects of ageing on mitochondria, including declines in mitochondrial content and bioenergetics, increased ROS emission, and perturbations of mitochondrial quality control (Fig. 1). Consequently, exercise training is an essential primary intervention to reduce many of the age-related declines in skeletal muscle mass and function that take place with an inactive lifestyle. However, levels of habitual physical activity steadily decline with age, and many older individuals fail to meet the physical activity guidelines outlined by the World Health Organisation [10]. Therefore, complementary interventions that promote the maintenance of skeletal muscle mitochondrial health and overall physical function with ageing are needed. While no current pharmacotherapies are effective in the treatment of sarcopenia, there have been intense research efforts focussed on the potential for supplements to improve mitochondrial function and promote muscle health and physical function in older individuals.

## Targeting the Mitochondria Through Supplements

Supplements can impact mitochondria through several mechanisms, including increases in mitochondrial biogenesis and bioenergetics, decreases in mitochondrial ROS emission and oxidative damage, and improvements in mitochondrial quality control (Table 1). The following section provides a contemporary evaluation of the evidence for the potential of several novel supplements to improve physical function in older individuals via their interactions with mitochondria. A thorough literature search of online databases was conducted to identify supplements that can improve physical function in older individuals. Supplements were included if there was evidence from human studies that this improvement may be mediated by mechanisms involving mitochondria. All authors agreed on the final list of supplements to be included in the review.

**Table 1:** The effects of supplements on skeletal muscle mitochondria and physical function in older individuals

Reference	Supplement protocol	Study design	Participant characteristics	Effects on skeletal muscle mass/physical function	Actions on skeletal muscle mitochondria	Alternate mechanisms
MitoQ						
Bispham et al. [76]	20 mg/day for 6 weeks	Randomised, placebo-controlled, crossover design	18 healthy males and females aged 60–79 years	Improved leg extension power	Not assessed	
Pham et al. [74]	20 mg/day for 6 weeks	Randomised, crossover design	20 healthy males aged 50 ± 1 years	Not assessed	Decreased mitochondrial H_2_O_2_ emission	Increased skeletal muscle catalase expression
Broome et al. [83]	20 mg/day for 10 days before acute exercise trial and during 3 weeks of high-intensity interval training	Randomised, placebo-controlled, parallel design	23 healthy males aged 44 ± 7 years	Augmented training-induced improvements in peak cycling power output	Augmented acute- exercise-induced increases in PGC-1α mRNA expression No effect of MitoQ on training-induced increases in skeletal muscle citrate synthase activity	Augmented acute exercise-induced increases in VEGF mRNA expression
Broome et al. [146]	20 mg/day for 6 weeks	Randomised, placebo-controlled, crossover design	19 male cyclists aged 44 ± 4 years	Improved 8 km cycling time trial performance	Not assessed	Attenuated acute exercise-induced increases in plasma F_2_-isoprostanes
Urolithin A						
Andreux et al. [87]	250, 500 and 1000 mg/day for 4 weeks	Randomised, placebo-controlled, crossover design	60 healthy males and females aged 61–85 years	Not assessed	500 and 1000 mg/day urolithin A increased markers of mitochondrial biogenesis in skeletal muscle	
Liu et al. [88]	1000 mg for 4 months	Randomised, placebo-controlled, parallel design	66 males (*n* = 16) and females (*n* = 50) aged 72 ± 5 years	Improved muscle endurance of the first dorsal interosseus No effect of urolithin A on the 6-min walk test	No effect of urolithin A on maximal ATP production	Decreased circulating inflammatory markers
Singh et al. [89]	500 and 1000 mg/day for 4 months	Randomised, placebo-controlled, parallel design	88 males (*n* = 32) and females (*n* = 56) aged 51 ± 7 years (500 mg group) and 52 ± 6 years (1000 mg group)	Improved leg muscle strength and clinically relevant improvements in the 6-min walk test and $\dot{V}$O_2peak_ No effect of urolithin A on peak cycling power output	Increased markers of mitochondrial biogenesis and mitophagy	Decreased circulating inflammatory markers
Omega-3 polyunsaturated fatty acids (n-3 PUFAs)						
Da Boit et al. [99]	2.1 g EPA/day and 0.6 g DHA/day for 18 weeks	Randomised, placebo-controlled, parallel design	50 males (*n* = 27) and females (*n* = 23) aged 71 ± 5 years (males) and 71 ± 3 years (females)	Augmented resistance training-induced increases in maximal isometric torque and improvement in muscle quality in females but not males	Not assessed	Augmented resistance training-induced decrease in plasma triglycerides in both sexes
Smith et al. [100]	1.86 g EPA and 1.50 g DHA/day for 6 months	Randomised, placebo-controlled, parallel design	44 healthy males (*n* = 15) and females (*n* = 29) aged 69 ± 7 years (placebo) and 68 ± 5 years (n3-PUFA)	Increased thigh muscle volume, handgrip strength, and one-repetition maximum strength	Not assessed	
Kunz et al. [101]	2.7 g EPA and 1.2 g DHA/day for 6 months	Randomised, placebo-controlled, parallel design	63 males (*n* = 29) and females (*n* = 34) aged 71 ± 5 years	Increased one-repetition maximum strength	No effect of n3-PUFAs on mitochondrial respiration, ATP production or ROS emission	Modest attenuation of the muscle protein synthesis response to acute exercise in n3-PUFA group
Murphy et al. [103]	0.8 g EPA and 1.1 g DHA/day combined with leucine-enriched protein for 24 weeks	Randomised, placebo-controlled, parallel design	107 males (n = 52) and females (n = 55) with low muscle mass aged ≥ 65 years	Decreased leg flexion strength and no effect on lean mass, handgrip strength, knee extension strength, physical performance, or muscle protein synthesis	Not assessed	
Bischoff-Ferrari et al. [104]	0.33 g EPA and 0.66 g DHA/day	Randomised, placebo-controlled, parallel design	1,900 males and females aged 75 ± 4 years	No effect on short physical performance battery	Not assessed	
Lalia et al. [105]	2.7 g EPA and 1.2 g DHA/day for 4 months	Pre-post study design	12 males (*n* = 5) and females (*n* = 7) aged 76 ± 5 years	Increased postabsorptive and post-exercise muscle protein synthesis	Decreased mitochondrial ROS emission No effect of n3-PUFAs on mitochondrial respiration	
Glycine and N-acetylcysteine (GlyNAC)						
Kumar et al. [112]	100 mg/kg/day glycine and N-acetylcysteine for 16 weeks	Randomised, placebo-controlled, parallel design	12 males (*n* = 8) and females (*n* = 4) aged 71 ± 4 years	Increased gait speed and muscle strength	Increased expression of mitochondrial regulators of biogenesis, energy metabolism, and mitophagy Increased fat oxidation and decreased glucose oxidation measured by indirect calorimetry	Decreased plasma F_2_-isoprostanes and markers of inflammation
Kumar et al. [113]	1.3 mmol/kg/day glycine and 0.81 mmol/kg/day cysteine given as N-acetylcysteine for 24 weeks	Pre-post study design	8 males (*n* = 3) and females (*n* = 5) aged 70–80 years	Improved gait speed, 6-min rapid walk test score, and grip strength Decreased muscle protein breakdown	Increased fat oxidation and decreased glucose oxidation measured by indirect calorimetry	Decreased plasma F_2_-isoprostanes and markers of inflammation
Kumar et al. [114]	1.3 mmol/kg/day glycine and 0.83 mmol/kg/day cysteine given as N-acetylcysteine for 12 weeks	Pre-post study design	8 males (*n* = 6) and females (*n* = 2) aged 45–65 years with human immunodeficiency virus	Improved gait speed, grip strength, chair-rise test score, and 6-min walk test score	Increased expression of mitochondrial regulators of energy metabolism and mitophagy Increased fat oxidation and decreased glucose oxidation measured by indirect calorimetry	Decreased plasma F_2_-isoprostanes and markers of inflammation
Sekhar [115]	1.33 mmol/kg/day glycine and 0.81 mmol/kg/day cysteine given as N-acetylcysteine for 14 days	Pre-post study design	12 adults with type 2 diabetes aged 51 ± 4 years	Not assessed	Increased fat oxidation and decreased glucose oxidation measured by indirect calorimetry	Decreased HOMA-IR and plasma free fatty acid concentrations
Nicotinamide riboside (NR)						
Dolopikou et al. [130]	500 mg 2 h before testing	Randomised, placebo-controlled, crossover design	12 males aged 72 ± 1 year	Increased peak isometric knee extensor torque and fatigue resistance	Not assessed	Decreased urine F_2_-isoprostanes and increased erythrocyte glutathione levels
Elhassan et al. [131]	1000 mg/day for 21 days	Randomised, placebo-controlled, parallel design	12 males aged 70–80 years	No effect on hand-grip strength	No effect on mitochondrial bioenergetics Downregulation of gene sets associated with energy metabolism	Decreased levels of circulating inflammatory cytokines
Dollerup et al. [132]	2000 mg/day for 12 weeks	Randomised, placebo-controlled, parallel design	40 insulin-resistant males aged 40–70 years	Not assessed	No effect on mitochondrial content or respiration	
Remie et al. [133]	1000 mg/day for 6 weeks	Randomised, placebo-controlled, crossover design	13 overweight or obese males (*n* = 6) and females (*n* = 7) aged 59 ± 5 years	Not assessed	No effect on mitochondrial respiration	
Nicotinamide mononucleotide (NMN)						
Pencina et al. [126]	1000 mg/day for 28 days	Randomised, placebo-controlled, parallel design	30 overweight or obese males (n = 16) and females (n = 14) aged 45 years or older	No effect on muscle strength, fatigue, aerobic capacity, or stair climbing power	Not assessed	
Yoshino et al. [127]	250 mg/day for 10 weeks	Randomised, placebo-controlled, parallel design	25 females with prediabetes	Not assessed	No effect on mitochondrial respiratory capacity	Improved insulin sensitivity
Igarashi et al	250 mg/day for 12 weeks	Randomised, placebo-controlled, parallel design	42 males aged 65 years or older	Improved gait speed and grip strength	Not assessed	

*PGC-1α* peroxisome proliferator-activated receptor gamma coactivator 1-alpha, *VEGF* vascular endothelial growth factor, *EPA* eicosapentaenoic acid, *DHA* docosahexaenoic acid

### MitoQ

Despite being a major source of cellular ROS, mitochondria are particularly sensitive to ROS-induced oxidative damage, and ageing is associated with increased skeletal muscle mitochondrial ROS emission in the presence of non-saturating ADP concentrations [46]. ROS production by mitochondria can lead to oxidative damage to mitochondrial proteins, membranes and DNA, impairing the ability of mitochondria to synthesise ATP and carry out their normal metabolic functions [66]. Furthermore, elevated ROS emission activates proteolytic pathways in skeletal muscle and oxidative stress has been implicated in the pathophysiology of age-related muscle atrophy [19]. Consequently, there has been growing interest in the potential for mitochondria-targeted antioxidants to attenuate age-related mitochondrial oxidative stress and maintain skeletal muscle health with ageing [67].

**Fig. 2:**
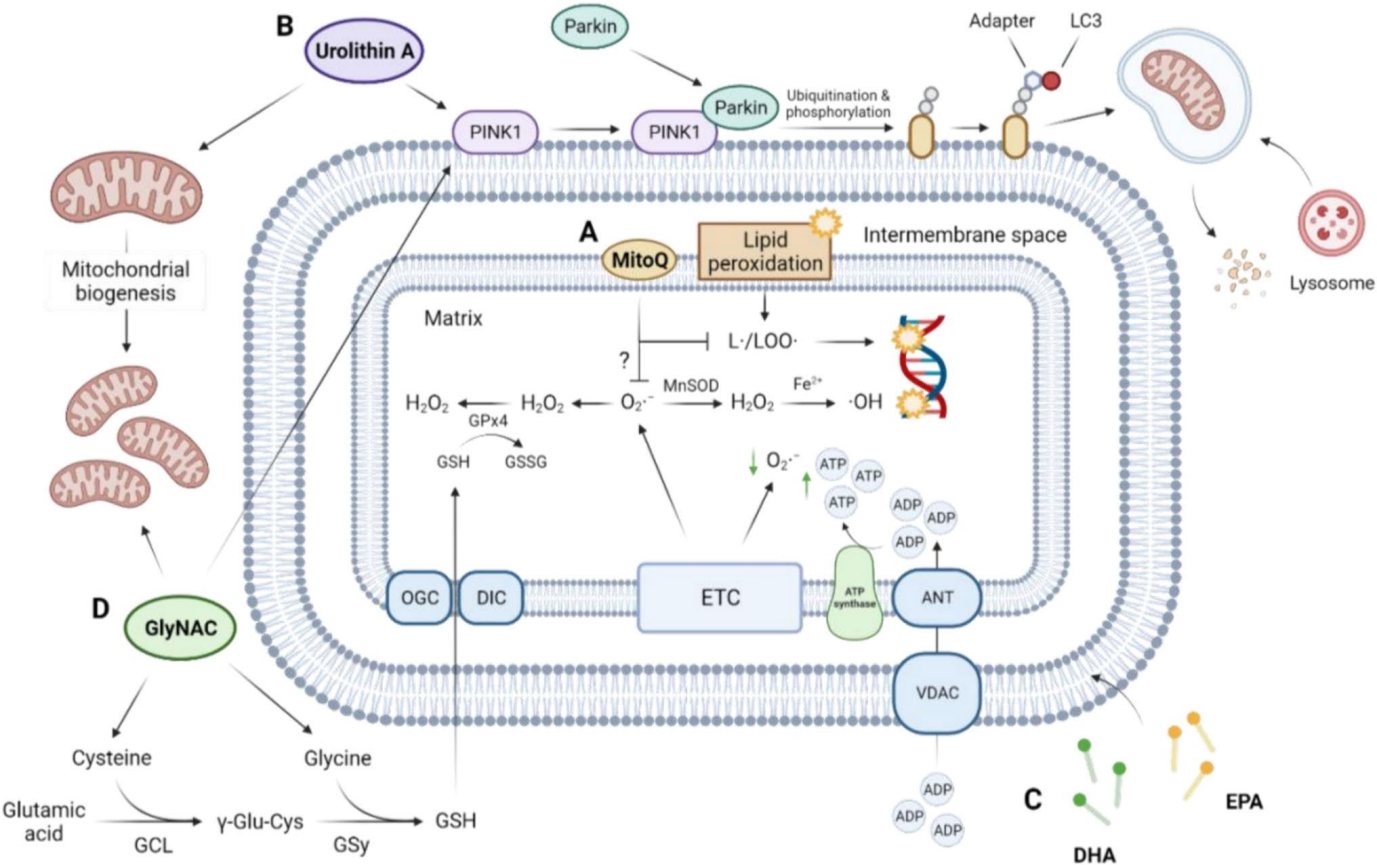
Supplements that promote physical function in ageing through their interactions with mitochondria. (**A**) MitoQ localises to the inner mitochondrial membrane and prevents the formation of lipid peroxidation products. MitoQ may also prevent mitochondrial superoxide production by interacting with proton motive forces and the coenzyme Q pool redox state. (**B**) Urolithin A activates mitochondrial biogenesis and PINK1-Parkin-mediated mitophagy. Parkin is recruited to PINK1 and ubiquitinates mitochondrial proteins. PINK1 then phosphorylates ubiquitin chains, leading to the accumulation of phospho-ubiquitinated mitochondrial proteins that serve as docking sites for adapter proteins that bind LC3. LC3 allows the formation of a phagosome membrane around mitochondria, which then fuses with and is broken down by lysosomes. (**C**) Incorporation of n-3 PUFAs EPA and DHA into mitochondrial membranes maintains mitochondrial energetics and decreases ROS emission. (**D**) GlyNAC provides precursors for the restoration of cellular GSH levels. GSH enters the mitochondrial matrix via different carriers, particularly the OGC and the DIC, where it aids in the detoxification of ROS. GlyNAC also increases the expression of regulators of mitochondrial biogenesis and mitophagy. *PINK1* PTEN induced kinase 1, *L•* lipid radical, *LOO•* lipid peroxy radical, *H*_*2*_*O*_*2*_ hydrogen peroxide, *O*_*2*_*•*^−^ superoxide, *MnSOD* manganese-dependent superoxide dismutase, *•OH* hydroxyl radical, *GPx4* glutathione peroxide 4, *GSH* reduced glutathione, *GSSG* oxidised glutathione, *ATP* adenosine triphosphate, *ADP* adenosine diphosphate, *ANT* adenine nucleotide translocase, *VDAC* voltage-dependent anion channel, *OGC* 2-oxoglutarate carrier, *DIC* dicarboxylate carrier, ETC electron transport chain, *DHA* docosahexaenoic acid, *EPA* eicosapentaenoic acid, *GlyNAC* glycine and N-acetylcystine, *GCL* glutamate cysteine ligase, *Glu-Cys* γ-glutamylcysteine, *GSy* glutathione synthetase

Coenzyme Q10 (CoQ10) is an essential component of the mitochondrial electron transport chain with antioxidant properties. Studies investigating the efficacy of CoQ10 to improve physical function in older individuals have shown equivocal results [68], which may, in part, be related to its poor bioavailability [69]. Conversely, mitochondria-targeted coenzyme Q10 (Mitoquinone, MitoQ) consists of a ubiquinone moiety attached to a lipophilic cation, which enables it to pass through the plasma membrane and accumulate in mitochondria via the plasma and mitochondrial membrane potential [70]. Within mitochondria, MitoQ localises to the inner mitochondrial membrane where it acts as an antioxidant, primarily by preventing lipid peroxidation (Fig. 2A). Mitochondrial membranes are rich in phospholipids that are susceptible to ROS-induced peroxidation, and lipid peroxidation byproducts can also damage mtDNA. This ROS-induced damage to mitochondrial lipids and DNA may compromise the mitochondrial membrane structure, ultimately generating a chain reaction and further amplifying mitochondrial ROS. As such, the antioxidant properties of MitoQ may act to indirectly reduce mitochondrial ROS production by protecting mitochondrial components from oxidative damage. MitoQ may also affect mitochondrial superoxide production by interacting with membrane potential (MitoQ uptake into mitochondria decreases proton motive force) and the coenzyme Q pool redox state [71]. MitoQ has been safely administered as a single oral 160 mg dose [72] or as a daily dose up to 80 mg [73]. However, supplementation with MitoQ at doses over 40 mg is sometimes associated with mild gastrointestinal side effects in some individuals. As a result, most studies provide MitoQ at a dose of 20 mg/day. MitoQ supplementation has been shown to decrease mitochondrial ROS emissions, increase protein expression of the antioxidant enzyme catalase [74], and attenuate exercise-induced DNA damage in skeletal muscle [75], suggesting MitoQ is available to skeletal muscle. However, there are currently no published data on the extent to which supplementation increases MitoQ levels in human skeletal muscle.

Human studies that have investigated the effects of MitoQ supplementation on skeletal muscle mass and function in an ageing context are limited. Six weeks of daily 20 mg MitoQ supplementation improved leg-extension power in healthy middle-aged and older individuals [76]. However, the effects of MitoQ supplementation on skeletal muscle mitochondrial respiration and ROS emission were not determined in that study [76]. Six weeks of MitoQ supplementation (20 mg/day) decreased maximal mitochondrial H_2_O_2_ emission rates and increased catalase expression in skeletal muscle of middle-aged men [74]. Given that high ROS levels impair muscle contractile function [77], the improvements in physical function observed in older individuals following MitoQ supplementation may be related to a decrease in ROS levels in skeletal muscle. Despite observing decreases in skeletal muscle ROS emission, mitochondrial content and respiration were unaffected following 6 weeks of 20 mg/day MitoQ supplementation in middle-aged men [74]. The effects of MitoQ supplementation on mitochondrial function may be greater in individuals who have higher mitochondrial ROS emission and lower levels of mitochondrial function. Therefore, studies involving longer supplementation periods are needed to determine the long-term effects of MitoQ supplementation on mitochondrial and physical function in older adults.

As outlined in earlier sections of this review, exercise is crucial for maintaining muscle mass and function at all ages but becomes increasingly important with advancing age. Research into the mechanisms underlying the beneficial effects of exercise has identified ROS as key signalling molecules that regulate exercise adaptations in skeletal muscle. However, several ROS-stimulated exercise responses, including acute stress responses [78] and anabolic responses [79], are attenuated in aged skeletal muscle regardless of training status, which may blunt several of the health-promoting effects of exercise in older individuals. Age-related increases in mitochondrial ROS emission have been linked to the failure of ROS-stimulated exercise responses in skeletal muscle of older individuals [80]. It has been proposed that increased mitochondrial ROS emission in aged muscle stimulates retrograde mitonuclear communication to increase the expression of a range of cytoprotective proteins that protect against oxidative damage [80]. This adaptive response may modify the local cytosol redox environment to induce a more reductive state in key cysteines of specific signalling proteins. In support of this hypothesis, expression of both cytosolic and mitochondrial forms of the antioxidant enzyme thioredoxin is elevated in muscles from old mice compared to younger mice [81]. Furthermore, redox proteomic analysis of human skeletal muscle has shown that ageing is associated with decreased levels of cysteine oxidation in several proteins [82]. Shifting to a more reduced cytosolic environment may prevent the transient oxidation of redox-sensitive signalling proteins during exercise and attenuate health-promoting exercise adaptations. Given that age-related increases in mitochondrial ROS emissions have been linked to the failure of redox responses to exercise in older individuals, interventions that decrease mitochondrial ROS emission in older individuals may restore these responses. MitoQ supplementation has been shown to increase training-induced improvements in peak cycling power output in middle-aged men, which may be related to the augmentation of the skeletal muscle transcriptional coactivator peroxisome proliferator-activated receptor gamma coactivator 1-alpha (PGC-1α) and vascular endothelial growth factor (VEGF) mRNA following acute exercise [83]. Further studies are required to determine whether MitoQ supplementation can restore redox responses to exercise in aged skeletal muscle.

### Urolithin A

Urolithin A is a natural compound produced in the large intestine by gut bacteria from ingested ellagitannins and ellagic acid, which are polyphenols present in a variety of plant products and foods, including pomegranates, strawberries, raspberries and walnuts [84, 85]. However, the ingestion of ellagitannin-rich foods is not always sufficient to elevate circulating levels of urolithin A, as specific gut microbiota are needed to convert ellagitannin into urolithin A. Urolithin A was only detectable in the plasma of 12% of healthy participants screened, while supplementation with urolithin A bypasses the need for microbiome-mediated conversion of urolithin A precursors and results in significantly elevated urolithin A levels in healthy adults [86]. Specifically, pharmacokinetic data indicate urolithin A is bioavailable at doses ranging from 250 to 2,000 mg, has a relatively long half-life (t_1/2_ = 17–22 h), and can be detected in skeletal muscle following oral supplementation [87].

Recent studies in humans provide evidence that urolithin A supplementation has positive effects on physical function in older individuals. Four months of daily supplementation with 1000 mg urolithin A improved muscular endurance during repeated contractions in older adults who presented with average physical performance measures (defined as 6-min walk distance ≤ 550 m) and low-average maximal ATP synthesis rates (≤ 1.0 mM/s measured using magnetic resonance spectroscopy) [88]. Clinically relevant improvements in whole-body measures of physical performance (6-min walk test distance and V̇O_2peak_) have also been observed following urolithin A supplementation [89]. Improved lower-body muscle strength was observed following 4 months of daily supplementation with 500 mg urolithin A supplementation in overweight middle-aged individuals [89]. Improvements in muscle strength following urolithin A supplementation were associated with an enrichment of gene sets related to muscle contraction and ribosome in skeletal muscle biopsies [89], suggesting urolithin A may activate an ‘anabolic’ response in skeletal muscle. Ageing is associated with anabolic resistance, a blunted stimulation of muscle protein synthesis in response to common anabolic stimuli such as exercise and dietary protein [79]. Given that age-related anabolic resistance contributes to the progressive decline in muscle mass and strength, further research is required to establish whether activation of anabolic responses in skeletal muscle by urolithin A has beneficial effects on muscle mass and strength in older individuals.

Improvements in physical performance following urolithin A supplementation are consistently associated with improvements in markers of skeletal muscle mitochondrial health (Fig. 2B). Urolithin A supplementation decreases plasma acylcarnitines [87–89], suggesting a decrease in incomplete fatty acid (FA) oxidation. Consistent with this finding, urolithin A increases the expression of several mitochondrial genes and tricarboxylic acid (TCA) cycle and oxidative phosphorylation protein levels in skeletal muscle [87, 89]. Collectively, these data suggest that urolithin A stimulates mitochondrial biogenesis and improves mitochondrial FA metabolism in skeletal muscle. This premise is supported by proteomic analysis of human skeletal muscle revealing that urolithin A supplementation increases markers of PTEN-induced kinase 1 (PINK1)/Parkin-mediated mitophagy [89].

**Fig. 3:**
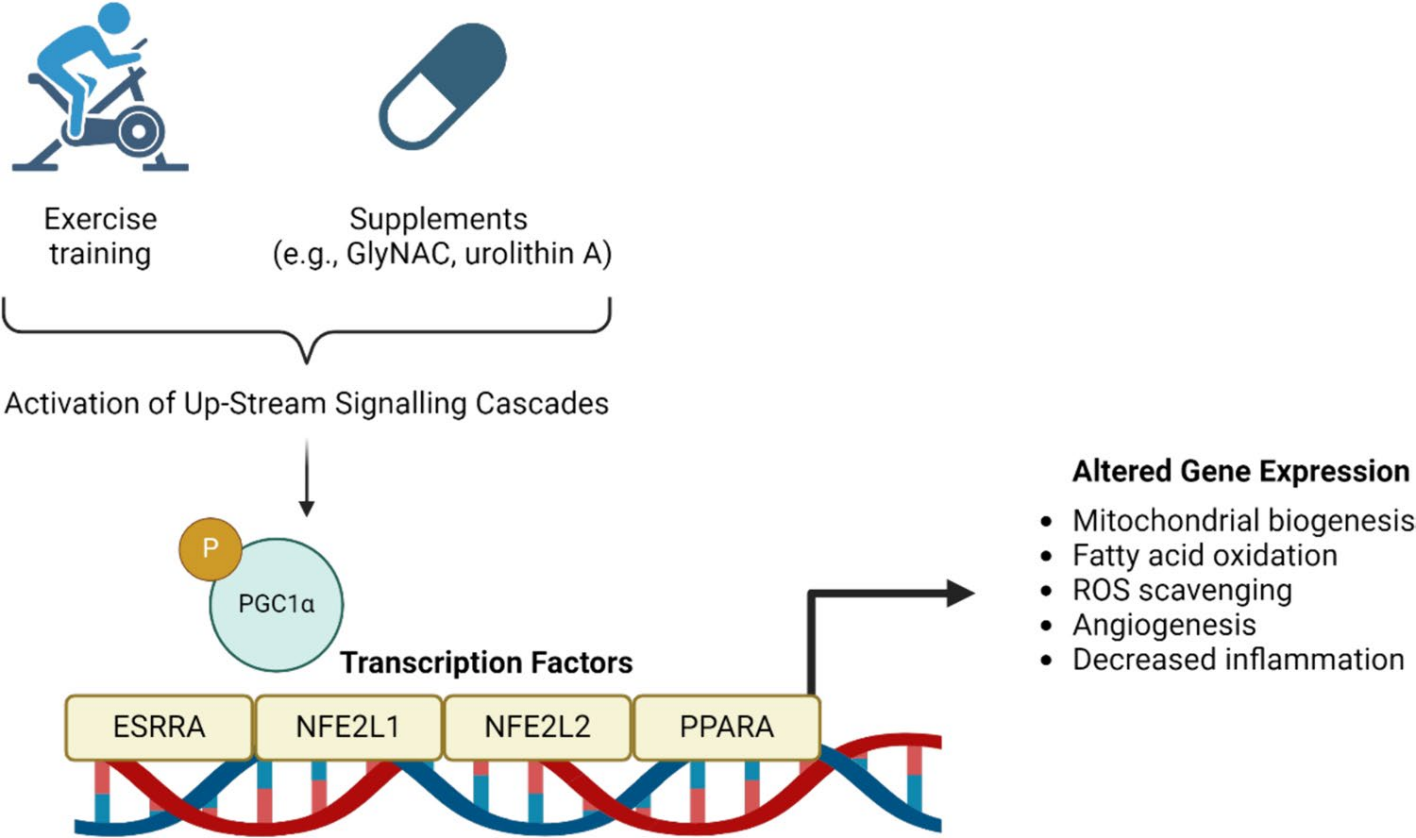
Both exercise and supplements such as urolithin A or GlyNAC activate cell signalling networks in skeletal muscle that target the peroxisome proliferator-activated receptor-γ coactivator 1α isoform 1 (PGC-1α), which may confer protective effects on skeletal muscle during ageing. Specifically, classical signalling kinases such as the 5’AMP activated protein kinase (AMPK) [147] or p38 MAPK [148] can phosphorylate PGC-1α, stabilizing the protein and promoting cellular accumulation. Within the nucleus, PGC-1α can bind to a host of transcription factors, including nuclear estrogen related receptors (ERRs) [149], respiratory factors (NRF) 1 and 2 [150], as well as peroxisome proliferator-activated receptor receptors (PPARs) [151]. This promotes the development of a more oxidative phenotype in muscle through mitochondrial biogenesis and increased expression of proteins associated with lipid uptake, transport, and metabolism [151, 152]. Furthermore, PGC-1α can exert an anti-inflammatory role in muscle by regulating the expression of proinflammatory cytokines such as interleukin-6 and tumor necrosis factor-alpha (TNF-α) [153, 154] and decreasing ROS production in part via the induction of genes that activate mitochondrial uncoupling proteins [155, 156]. *GlyNAC* glycine and N-acetylcysteine, *PGC-1α* peroxisome proliferator-activated receptor-γ coactivator 1α isoform, *1ESRRA* estrogen-related receptor α, *NFE2L1* nuclear factor erythroid-derived 2 like-1, *NFE2L2* nuclear factor erythroid‐derived 2‐like 2, *PPARA*,peroxisome proliferator activated receptor alpha, *ROS* reactive oxygen species

In addition to its benefits on mitochondrial health, urolithin A also decreases plasma biomarkers of inflammation, including C-reactive protein (CRP) and inflammatory cytokines [88, 89]. Therefore, urolithin A may offer a potential dual benefit to muscle health during ageing by improving both mitochondrial function and attenuating age-related chronic inflammation. Urolithin A induction of mitophagy could mediate its anti-inflammatory effect, as removing dysfunctional mitochondria reduces the production of ROS and the release of mtDNA and cardiolipins, both known triggers of inflammatory responses [90]. Alternatively, urolithin A-mediated reduction of inflammatory markers may contribute to blunting their negative regulation of mitochondrial biogenesis effectors, such as the PGC-1α and sirtuin 1 (SIRT1), thereby allowing the generation of new mitochondria [90]. PGC-1α upregulates mitochondrial and metabolic gene expression, synchronizes several exercise-associated aspects of muscle plasticity and function, while concomitantly suppressing a broad inflammatory response [91]. Therefore, upregulation of PGC-1α in skeletal muscle following both exercise and supplementation with urolithin A may confer several local and whole-body effects, conferring protection against age-related declines in skeletal muscle mass and physical function [92] (Fig. 3).

### Omega-3 Polyunsaturated Fatty Acids

The omega-3 polyunsaturated FA (n-3 PUFAs) eicosapentaenoic acid (EPA) and docosahexaenoic acid (DHA) are critical for the structure and function of biological membranes. While n-3 PUFAs can be obtained following the consumption of fatty fish such as salmon, herring, mackerel, tuna and sardines, supplementation with EPA and DHA results in incorporation of these n-3 PUFAs into phospholipid membranes within skeletal muscle [93–95]. Population recommendations for EPA and DHA intake are typically 250–500 mg/day for EPA and DHA combined. However, studies investigating the effects of n-3 PUFA supplementation on physical function typically use higher doses (intakes of up to 5 g/day), which are difficult to obtain from food sources. While this dose is considered safe for human supplementation, the minimal n-3 PUFA regimen required to improve physical function is currently unknown.

A growing body of evidence suggests that n-3 PUFA supplementation positively impacts skeletal muscle anabolism and strength gains [96]. Eight weeks of n-3 PUFA supplementation (1.9 g/day EPA and 1.5 g/day DHA) increases rates of muscle protein synthesis in young, middle-aged and older adults in response to a hyperaminoacidemic-hyperinsulinemic clamp [97, 98]. Furthermore, n-3 PUFA supplementation (2.1 g/day EPA and 0.6 g/day DHA) amplified the improvements in muscle strength and quality observed in older women, but not men, following 18 weeks of resistance exercise training [99]. n-3 PUFA supplementation has also been shown to improve skeletal muscle mass and strength in older men and women [100, 101] and attenuate immobilisation-induced declines in skeletal muscle mass in healthy young women [102]. Collectively, these observations indicate that n-3 PUFAs may protect against age-related declines in muscle mass and function and muscle atrophy during acute periods of disuse (e.g., before and after elective surgery). However, while some studies report improvements in muscle anabolism and physical function following n-3 PUFA supplementation, this is not a universal finding. Six months of n-3 PUFA supplementation (1.6 g/day EPA and 2.3 g/day DHA) failed to improve lean mass, muscle strength or whole-body physical performance in 107 older males and females [103]. Similarly, chronic (3 years) n-3 PUFA supplementation (0.3 g/day EPA and 0.7 g/day DHA) failed to improve whole-body physical performance when taken alone or in combination with strength training in a cohort of 2,000 healthy men and women aged 70 years and over [104]. These conflicting results may, in part, be explained by differences in the dose administered, or by differences in baseline physical function and nutrition status of the participants as individuals with reduced physical function and/or lower protein intakes may experience greater benefits following n-3 PUFA supplementation.

The mechanisms underlying improved muscle anabolism following n-3 PUFA supplementation are unknown but increasing evidence suggests that these effects may be mediated by mitochondria (Fig. 2C). Incorporation of n-3 PUFAs into phospholipid membranes impacts the organisation and function of membrane proteins, which is important in mitochondria where electron transfer is tightly coupled between the complexes of the electron transport chain embedded in the inner mitochondrial membrane. Sixteen weeks of n-3 PUFA supplementation (2.7 g/day EPA and 1.2 g/day DHA) decreased mitochondrial H_2_O_2_ emissions in skeletal muscle of older individuals [105], suggesting n-3 PUFA supplementation may be an effective strategy to attenuate age-related increases in skeletal muscle mitochondrial ROS production and oxidative stress. Improvements in ADP sensitivity have also been observed in skeletal muscle of young individuals following n-3 PUFA supplementation [93, 106]. This is important from an ageing perspective as mitochondrial ADP sensitivity is decreased in skeletal muscle of older individuals, resulting in increased mitochondrial ROS emission [46]. Age-related anabolic resistance and loss of muscle mass and strength have also been linked to elevated skeletal muscle mitochondrial ROS levels [80, 107]. Therefore, increasing mitochondrial ADP sensitivity and reducing mitochondrial ROS levels may be one mechanism through which n-3 PUFA supplementation improves muscle mass and strength in older individuals.

Supplementation with n-3 PUFAs (3 g/day EPA and 2 g/day DHA) attenuates reductions in mitochondrial protein content and respiration independent of changes in mitochondrial H_2_O_2_ emissions after 2 weeks of single-leg immobilisation in young women [106]. These mitochondrial responses were associated with an attenuated loss of MRI-measured skeletal muscle volume and leg lean mass [102]. Whether the effects of n-3 PUFA supplementation on mitochondrial protein content and respiration are a cause or a consequence of changes in muscle mass is currently unknown. However, the loss of muscle mass during periods of disuse is preceded by reductions in mitochondrial content [108]. Given that cytosolic protein synthesis is an energetically costly process, and mitochondria are the primary source of cellular energy, retrograde signalling mechanisms may exist to maintain the balance between cytosolic and mitochondrial protein synthesis/bioenergetics [109]. Therefore, maintenance of mitochondrial bioenergetics during disuse may sustain mitochondrial and cytosolic protein transcription and translation [96]. Such a hypothesis has not been tested in humans and requires further investigation.

### Glycine and N-Acetylcysteine (GlyNAC)

Glutathione (GSH) is the most abundant endogenous intracellular antioxidant. GSH donates reducing equivalents to neutralize H_2_O_2_ to water in a reaction where GSH itself becomes oxidized to GSSG. Therefore, GSH exists and cycles between a reduced (GSH) and oxidised (GSSG) state to maintain an optimal cellular redox state. GSH is a tripeptide composed of three amino acids: glycine, cysteine and glutamic acid. In older adults, impaired GSH synthesis caused by decreased availability of glycine and cysteine (but not glutamic acid) contributes to the development of GSH deficiency, leading to increased levels of oxidative stress [110]. GSH deficiency cannot be corrected through oral GSH supplementation as GSH is digested in the gut. However, daily oral supplementation with GlyNAC, a combination of GSH precursor amino acids glycine and cysteine (provided as N-acetylcysteine, NAC), can correct glycine and cysteine deficiencies, restore impaired GSH synthesis, and increase intracellular GSH concentrations in older individuals [110–112]. There is now widespread acceptance that ROS play an important role in cell signalling and that antioxidant supplementation is a double-edged sword: too much can be as detrimental as too little. Therefore, GlyNAC supplementation is an attractive intervention as it provides precursor amino acids to allow cells to autoregulate their own GSH synthesis based on cellular need.

GlyNAC supplementation (12–24 weeks, 100 mg/kg/day glycine and cysteine) improved gait speed, muscle strength and exercise capacity in older individuals [112, 113] and patients with HIV who develop the onset of geriatric conditions at an early age [114]. GlyNAC supplementation has also been shown to attenuate age-related increases in muscle protein breakdown [113], which has important implications for the maintenance of muscle mass during ageing. Multiple factors could be responsible for the observed improvements in physical function following GlyNAC supplementation as GlyNAC promotes positive effects on mitochondria, oxidative stress and inflammation, all of which interact and contribute to the decline in skeletal muscle mass and function occurring with ageing (Fig. 2D). Improvements in physical function following GlyNAC supplementation are consistently associated with increases in fat oxidation measured using calorimetry [112–115] and increases in protein expression of regulators of mitochondrial biogenesis (PGC-1α) and energy metabolism including mitochondrial FA transport (CPT1b), β-oxidation (HADHA), electron complexes (I, II, III, IV), ATP synthesis (ATP synthase F1 subunit alpha; ATP5A), and mitophagy (PINK1) [112, 114]. While evidence from human studies suggests that GlyNAC improves physical function in ageing via interactions with mitochondria, these results should be interpreted with caution as the majority of data derive from pilot trials with small sample sizes and no placebo group [113–115].

### NAD^+^ Precursors

Nicotinamide adenine dinucleotide (NAD^+^) is a crucial universal cofactor. In its reduced form, NADH fuels oxidative phosphorylation by supplying the mitochondrial electron transport chain with electrons from glycolysis and the TCA cycle. NAD^+^ also has an important role as a substrate in multiple enzymatic processes. NAD^+^ is replenished through three pathways: the Preiss-Handler pathway, the kynurenine pathway and the salvage pathway [116]. The Preiss-Handler pathway converts nicotinic acid (NA) through nicotinic acid mononucleotide (NAMN) and nicotinic acid adenine dinucleotide (NAAD) to NAD^+^. The kynurenine pathway begins with tryptophan and converges with the Preiss-Handler pathway at the level of NAMN. Nicotinic acid riboside (NAR) also converges with the Preiss-Handler pathway at the level of NAMN. In the salvage pathway, NAM is converted to NAD^+^ through nicotinamide mononucleotide (NMN). Ageing is associated with a systemic decrease in NAD^+^ levels [116], which may contribute to declines in mitochondrial and physical function. As a result, there has been growing interest in the potential for supplementation with NAD^+^ precursors, including nicotinamide riboside (NR), NMN and NA to increase NAD^+^ levels and attenuate age-related pathology.

NR is the most well studied of all the NAD^+^ precursors [117], with its uptake mediated by the nucleoside transporter protein family [118]. Once inside the cell, NR can be phosphorylated by the NR kinases (NRK1 and NRK2) [119] or be deribosylated by the purine nucleoside phosphorylase to become NAM [120]. In principle, both conversions allow the production of NAD^+^ via entry into the salvage pathway. However, animal studies provide evidence that few tissues are exposed to large amounts of NR since the majority of orally administered NR is converted to NAM in the small intestine and then to NA by the gut microbiota [121–123]. The small amount of orally administered NR that enters the blood is rapidly converted to NAM in whole blood, meaning NR is not easily detectable in blood following oral administration [124]. Acute supplementation with 250 mg and chronic supplementation with between 100 and 2,000 mg NR per day for between 7 days and 5 months increased NAD^+^ and a range of its related metabolites in whole blood and peripheral blood mononuclear cells (PBMCs) [125–130]. However, to date, there is no evidence that oral NR increases muscle NAD^+^ levels, which likely explains why the majority of studies investigating the effects of NR supplementation on mitochondrial content and respiration have failed to show positive results [131–134]. Despite this, acute NR supplementation has been shown to increase peak isometric torque and attenuate fatigue in older individuals [130]. Given that high ROS levels impact skeletal muscle contractile function [77, 135], the improvements in physical function observed in that study may be related to improvements in redox homeostasis as NR supplementation decreased urine F_2_-isoprostanes and increased erythrocyte glutathione levels in the older group [130]. Further research is needed to elucidate the mechanisms through which NR improves physical function in older individuals as these effects appear to be unrelated to the capacity for oral NR supplementation to impact skeletal muscle NAD^+^ levels.

Oral supplementation with the NAD^+^ precursor NMN increases NAD^+^ and its metabolites in blood [126, 127, 136]. However, there is currently no evidence that NMN supplementation increases NAD^+^ levels in human skeletal muscle. Despite this, several studies have investigated the effect of NMN on physical function. Twelve weeks of daily 250 mg NMN supplementation improved walking speed and grip strength in older men [136]. In contrast, shorter periods of NMN supplementation (4–10 weeks) failed to impact muscle strength, fatigue and V̇O_2peak_ in individuals with prediabetes or middle-aged and older adults with overweight/obesity. These observations may be explained by the failure of NMN to impact muscle mitochondrial oxidative capacity [126, 127]. Taken together, the results from studies investigating the effect of oral NMN on physical function are conflicting, and further research is required to elucidate potential mechanisms by which NMN acts on skeletal muscle to exert any beneficial effect, if present. This is particularly important given that oral NMN supplementation has not been shown to impact muscle NAD^+^ levels.

NA can be incorporated into the NAD^+^ pool in various tissues via the Preiss-Handler pathway, but synthesis of NAD^+^ from NA in muscle appears to be low or non-existent [124, 137]. However, 10 months of NA treatment (escalating dose starting at 250 mg/day and reaching 1 g/day by 3 months) in patients with mitochondrial myopathy and NAD^+^ deficiency restored muscle NAD^+^ levels, increased mitochondrial respiratory enzyme activity and mitochondrial mass, and improved muscle strength and walking speed [138]. Results from that investigation [138] suggest that human trials involving NAD^+^ precursors may be of benefit to individuals with NAD^+^ deficiency, but further studies are needed in a variety of clinical populations to determine the effects of NA supplementation on muscle NAD^+^ levels and mitochondrial and physical function.

## Application to Master Athletes

While most older adults reduce their levels of habitual physical activity with advancing age, master athletes are a unique subpopulation in that they continue to maintain high levels of physical activity late in life, including structured and performance-driven exercise training. Master athletes exhibit remarkable physical function relative to their sedentary counterparts [139]. Furthermore, some master athletes even display muscular strength, performance, myofiber properties and function comparable with young, healthy individuals [140]. However, despite completing high levels of physical training that partially protect against the decline in musculoskeletal and cardiorespiratory health that occurs with ageing, master athletes still experience lower levels of performance compared to their young, trained counterparts [139]. Over 60% of master athletes report using nutritional supplements [141, 142], suggesting this population is aware of the potential benefits of such interventions to mitigate age-related declines in health and performance. Given that master athletes exhibit age-related declines in performance [139], attenuated adaptive responses [78, 143], and impaired recovery [144], the supplements discussed in this review could have ergogenic effects in this population through several mechanisms, including improvements in mitochondrial function, improved adaptive responses [83, 105], and decreased muscle damage/inflammation [89, 112, 145]. While studies in master athletes are limited, 6 weeks of MitoQ supplementation has been shown to attenuate exercise-induced oxidative stress and improve cycling time trial performance in master cyclists (average age 44 years) [146]. Further studies are required to determine whether the supplements discussed in this review have performance benefits for master athletes.

## Conclusion

This review has highlighted the critical role of both aerobic- and strength-based exercise training in attenuating many of the deleterious effects of ageing on skeletal muscle mitochondria, including decreases in mitochondrial content and bioenergetics, increased mitochondrial ROS emission, and perturbations of mitochondrial quality control. While exercise training is essential for the attenuation of age-related declines in skeletal muscle mass and function, many older adults have low levels of habitual physical activity. Therefore, there is a need to identify complementary interventions that can improve physical function in ageing either independently or in synergy with exercise. There is both mechanistic and functional evidence to demonstrate that several supplements can improve physical function in older individuals through direct or indirect effects on skeletal muscle mitochondria. In this context, MitoQ, urolithin A, n-3 PUFAs and GlyNAC improve physical function in older individuals through several mechanisms including increases in mitochondrial biogenesis and bioenergetics, decreases in mitochondrial ROS emission and oxidative damage, and improvements in mitochondrial quality control. While there is some evidence that some NAD^+^ precursors can improve physical function in older individuals, such an outcome seems unrelated to and independent of changes in skeletal muscle mitochondrial function. While this review has summarised the evidence for supplements that improve mitochondrial function and skeletal muscle health in elderly humans, there are several compounds that have shown similar effects in preclinical models (e.g., mitochondrial-derived peptides and mitochondrial uncouplers). Future studies should investigate the safety and efficacy of these compounds with a view to extending the human health-span.

## Data Availability

Data sharing is not applicable to this article as no new data were created or analyzed in this study.
